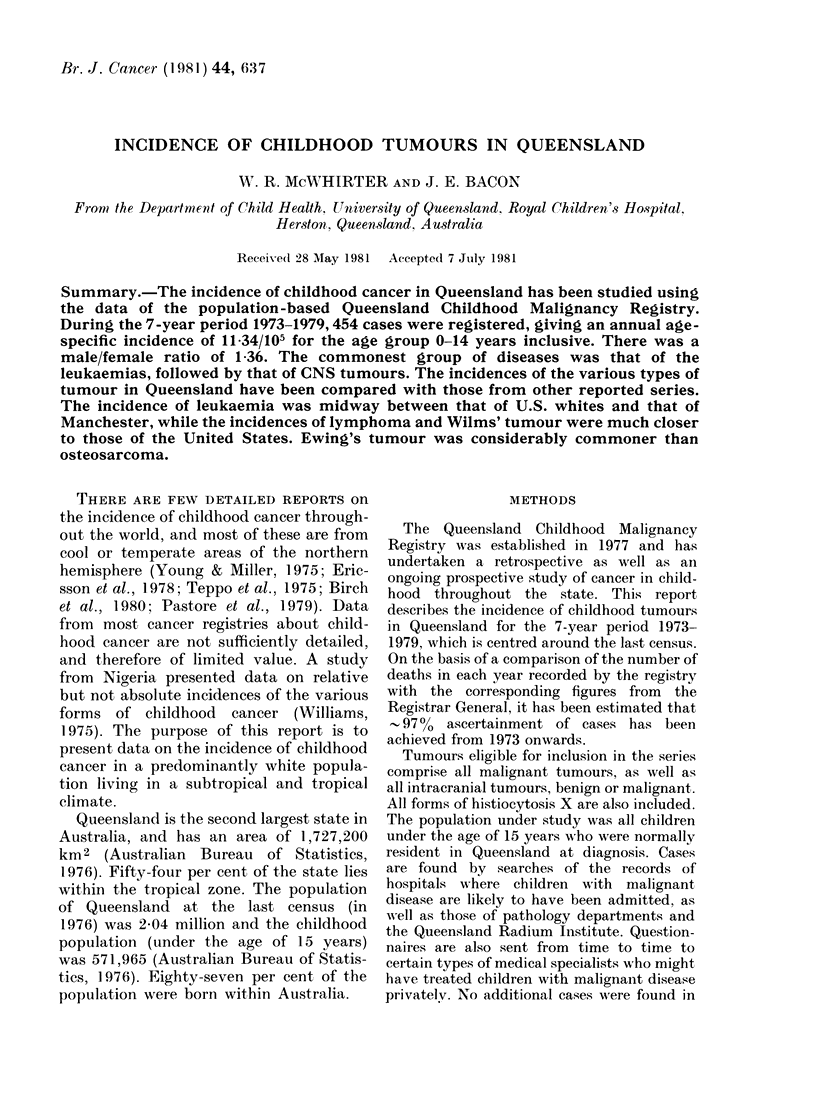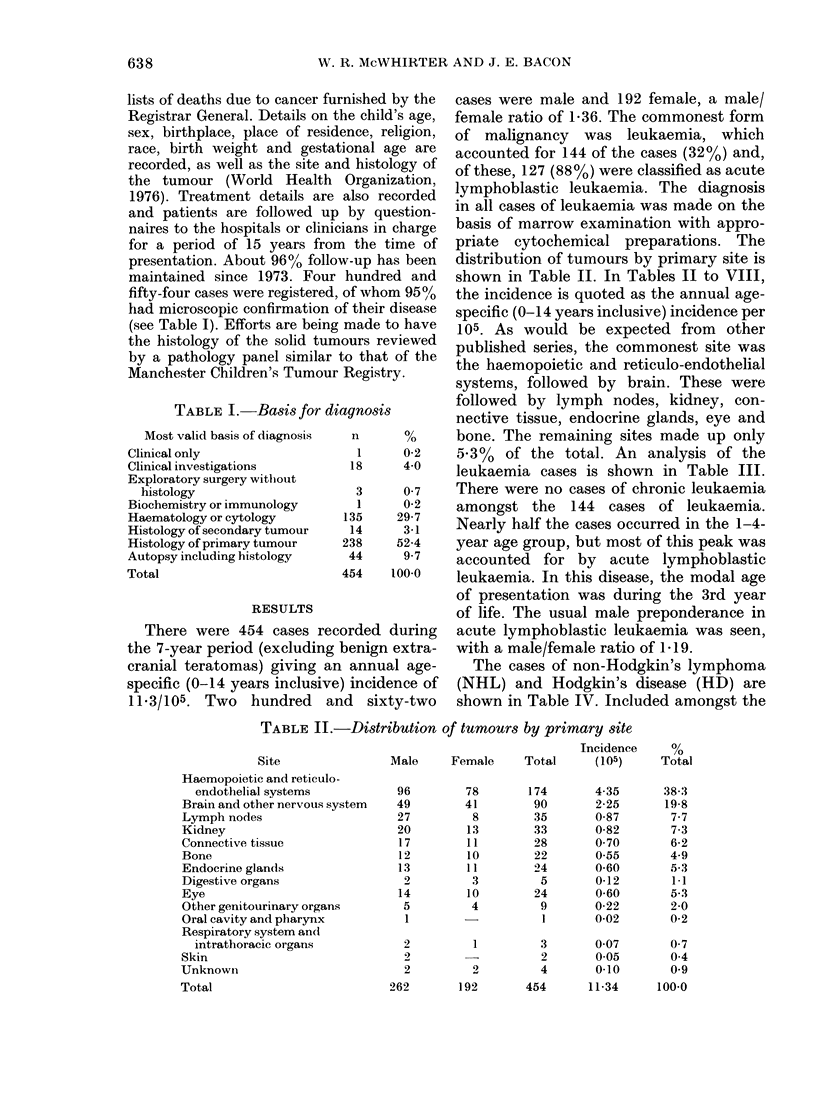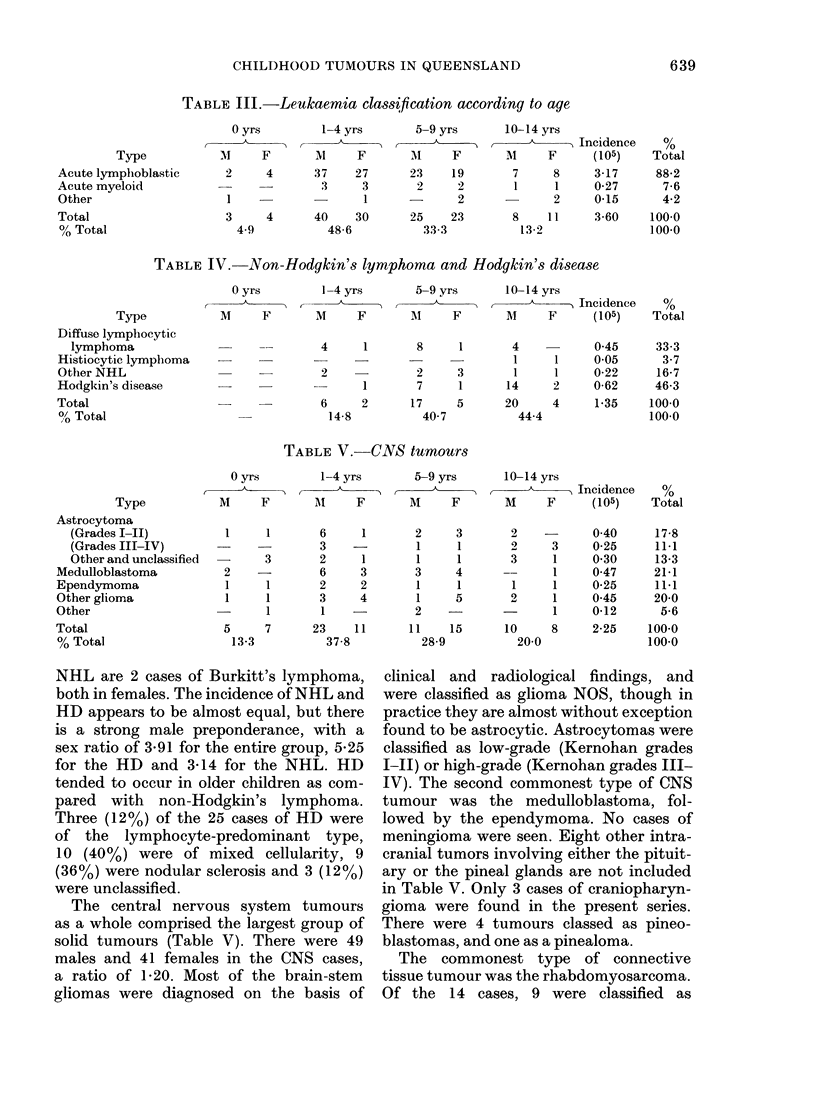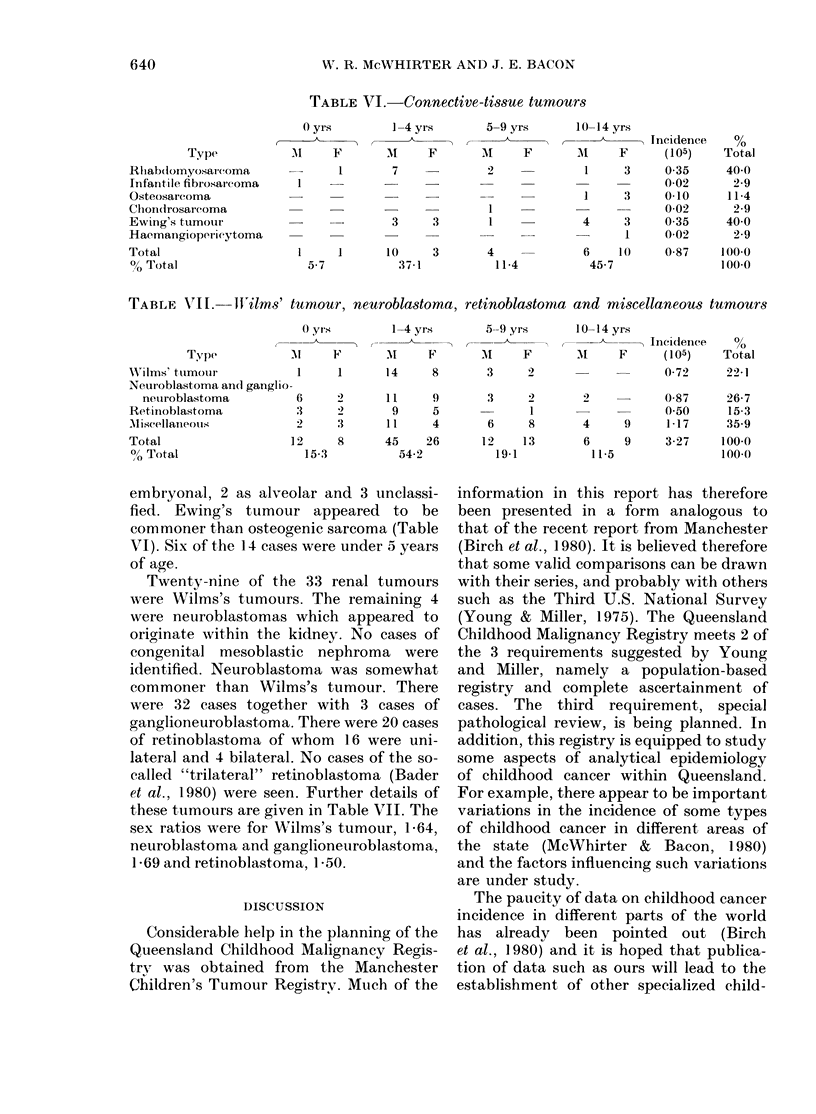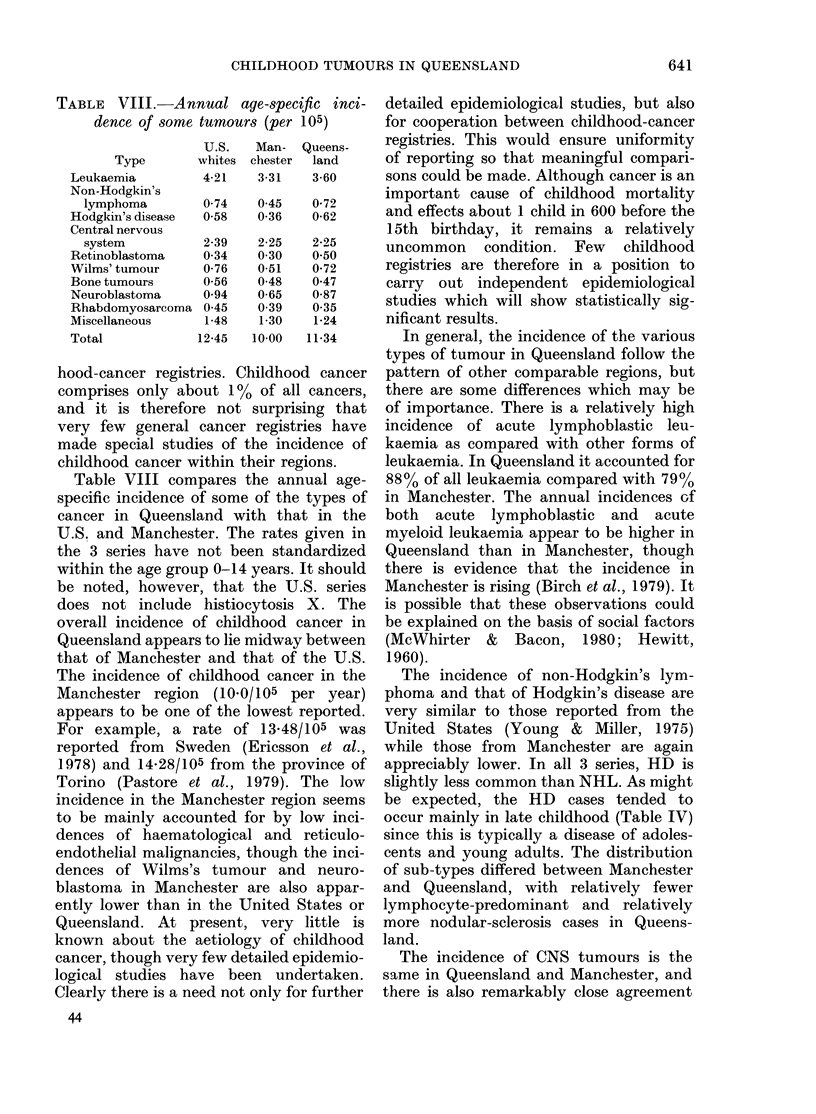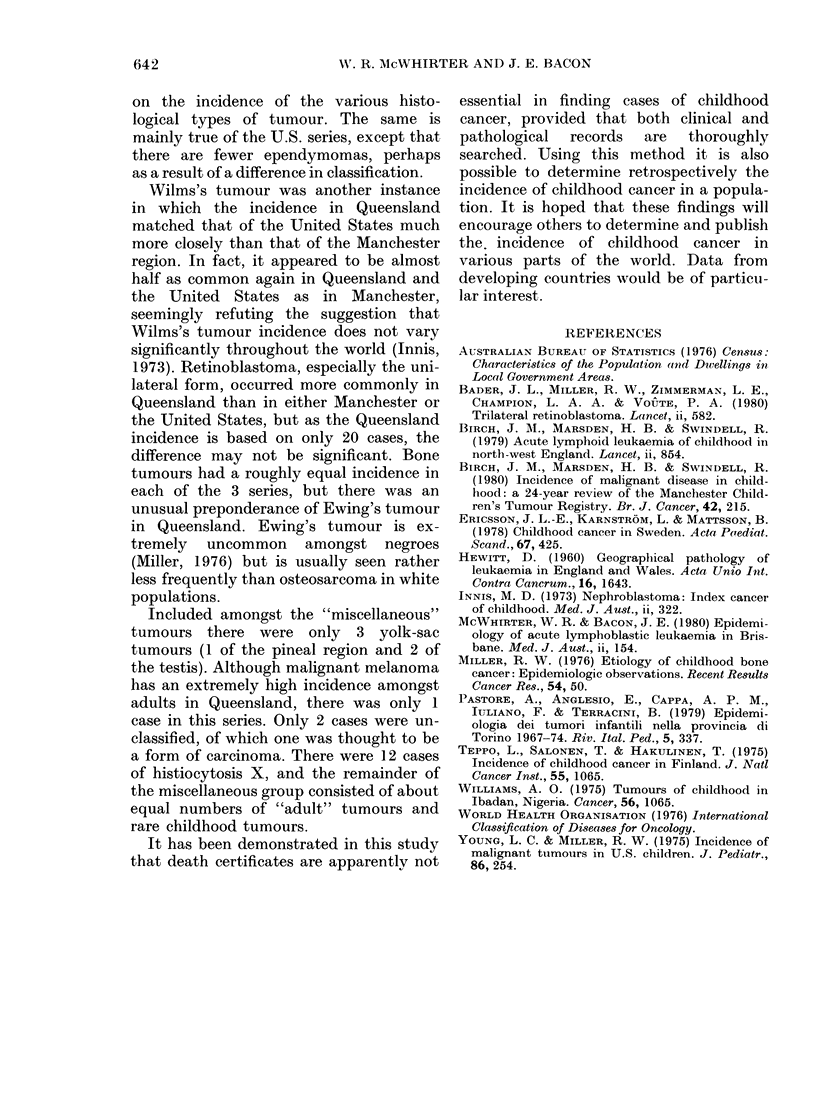# Incidence of childhood tumours in Queensland.

**DOI:** 10.1038/bjc.1981.248

**Published:** 1981-11

**Authors:** W. R. McWhirter, J. E. Bacon

## Abstract

The incidence of childhood cancer in Queensland has been studied using the data of the population-based Queensland Childhood Malignancy Registry. During the 7-year period 1973-1979, 454 cases were registered, giving an annual age-specific incidence of 11.34/10(5) for the age group 0-14 years inclusive. There was a male/female ratio of 1.36. The commonest group of diseases was that of the leukaemias, followed by that of CNS tumours. The incidences of the various types of tumour in Queensland have been compared with those from other reported series. The incidence of leukaemia was midway between that of U.S. whites and that of Manchester, while the incidences of lymphoma and Wilms' tumour were much closer to those of the United States. Ewing's tumour was considerably commoner than osteosarcoma.


*Br. J. Cancer* (1981) **44**, 637

### INCIDENCE OF CHILDHOOD TUMOURS IN QUEENSLAND

W. R. McWHIRTER AND J. E. BACON

*From the Department of Child Health, University of Queensland, Royal Children's Hospital, Herston, Queensland, Australia*

Received 28 May 1981 Accepted 7 July 1981

**Summary.—The incidence of childhood cancer in Queensland has been studied using the data of the population-based Queensland Childhood Malignancy Registry. During the 7-year period 1973–1979, 454 cases were registered, giving an annual age-specific incidence of 11·34/$10^5$ for the age group 0–14 years inclusive. There was a male/female ratio of 1·36. The commonest group of diseases was that of the leukaemias, followed by that of CNS tumours. The incidences of the various types of tumour in Queensland have been compared with those from other reported series. The incidence of leukaemia was midway between that of U.S. whites and that of Manchester, while the incidences of lymphoma and Wilms' tumour were much closer to those of the United States. Ewing's tumour was considerably commoner than osteosarcoma.**

THERE ARE FEW DETAILED REPORTS on the incidence of childhood cancer throughout the world, and most of these are from cool or temperate areas of the northern hemisphere (Young & Miller, 1975; Ericsson *et al.*, 1978; Teppo *et al.*, 1975; Birch *et al.*, 1980; Pastore *et al.*, 1979). Data from most cancer registries about childhood cancer are not sufficiently detailed, and therefore of limited value. A study from Nigeria presented data on relative but not absolute incidences of the various forms of childhood cancer (Williams, 1975). The purpose of this report is to present data on the incidence of childhood cancer in a predominantly white population living in a subtropical and tropical climate.

Queensland is the second largest state in Australia, and has an area of 1,727,200 km$^2$ (Australian Bureau of Statistics, 1976). Fifty-four per cent of the state lies within the tropical zone. The population of Queensland at the last census (in 1976) was 2·04 million and the childhood population (under the age of 15 years) was 571,965 (Australian Bureau of Statistics, 1976). Eighty-seven per cent of the population were born within Australia.

### METHODS

The Queensland Childhood Malignancy Registry was established in 1977 and has undertaken a retrospective as well as an ongoing prospective study of cancer in childhood throughout the state. This report describes the incidence of childhood tumours in Queensland for the 7-year period 1973–1979, which is centred around the last census. On the basis of a comparison of the number of deaths in each year recorded by the registry with the corresponding figures from the Registrar General, it has been estimated that ~97% ascertainment of cases has been achieved from 1973 onwards.

Tumours eligible for inclusion in the series comprise all malignant tumours, as well as all intracranial tumours, benign or malignant. All forms of histiocytosis X are also included. The population under study was all children under the age of 15 years who were normally resident in Queensland at diagnosis. Cases are found by searches of the records of hospitals where children with malignant disease are likely to have been admitted, as well as those of pathology departments and the Queensland Radium Institute. Questionnaires are also sent from time to time to certain types of medical specialists who might have treated children with malignant disease privately. No additional cases were found in lists of deaths due to cancer furnished by the Registrar General. Details on the child's age, sex, birthplace, place of residence, religion, race, birth weight and gestational age are recorded, as well as the site and histology of the tumour (World Health Organization, 1976). Treatment details are also recorded and patients are followed up by questionnaires to the hospitals or clinicians in charge for a period of 15 years from the time of presentation. About 96% follow-up has been maintained since 1973. Four hundred and fifty-four cases were registered, of whom 95% had microscopic confirmation of their disease (see Table I). Efforts are being made to have the histology of the solid tumours reviewed by a pathology panel similar to that of the Manchester Children's Tumour Registry.

TABLE I.—*Basis for diagnosis*

| Most valid basis of diagnosis | n | % |
|---|---|---|
| Clinical only | 1 | 0·2 |
| Clinical investigations | 18 | 4·0 |
| Exploratory surgery without histology | 3 | 0·7 |
| Biochemistry or immunology | 1 | 0·2 |
| Haematology or cytology | 135 | 29·7 |
| Histology of secondary tumour | 14 | 3·1 |
| Histology of primary tumour | 238 | 52·4 |
| Autopsy including histology | 44 | 9·7 |
| Total | 454 | 100·0 |

### RESULTS

There were 454 cases recorded during the 7-year period (excluding benign extracranial teratomas) giving an annual age-specific (0–14 years inclusive) incidence of $11{\cdot}3/10^5$. Two hundred and sixty-two cases were male and 192 female, a male/female ratio of 1·36. The commonest form of malignancy was leukaemia, which accounted for 144 of the cases (32%) and, of these, 127 (88%) were classified as acute lymphoblastic leukaemia. The diagnosis in all cases of leukaemia was made on the basis of marrow examination with appropriate cytochemical preparations. The distribution of tumours by primary site is shown in Table II. In Tables II to VIII, the incidence is quoted as the annual age-specific (0–14 years inclusive) incidence per $10^5$. As would be expected from other published series, the commonest site was the haemopoietic and reticulo-endothelial systems, followed by brain. These were followed by lymph nodes, kidney, connective tissue, endocrine glands, eye and bone. The remaining sites made up only 5·3% of the total. An analysis of the leukaemia cases is shown in Table III. There were no cases of chronic leukaemia amongst the 144 cases of leukaemia. Nearly half the cases occurred in the 1–4-year age group, but most of this peak was accounted for by acute lymphoblastic leukaemia. In this disease, the modal age of presentation was during the 3rd year of life. The usual male preponderance in acute lymphoblastic leukaemia was seen, with a male/female ratio of 1·19.

The cases of non-Hodgkin's lymphoma (NHL) and Hodgkin's disease (HD) are shown in Table IV. Included amongst the

TABLE II.—*Distribution of tumours by primary site*

| Site | Male | Female | Total | Incidence ($10^5$) | % Total |
|---|---|---|---|---|---|
| Haemopoietic and reticulo-endothelial systems | 96 | 78 | 174 | 4·35 | 38·3 |
| Brain and other nervous system | 49 | 41 | 90 | 2·25 | 19·8 |
| Lymph nodes | 27 | 8 | 35 | 0·87 | 7·7 |
| Kidney | 20 | 13 | 33 | 0·82 | 7·3 |
| Connective tissue | 17 | 11 | 28 | 0·70 | 6·2 |
| Bone | 12 | 10 | 22 | 0·55 | 4·9 |
| Endocrine glands | 13 | 11 | 24 | 0·60 | 5·3 |
| Digestive organs | 2 | 3 | 5 | 0·12 | 1·1 |
| Eye | 14 | 10 | 24 | 0·60 | 5·3 |
| Other genitourinary organs | 5 | 4 | 9 | 0·22 | 2·0 |
| Oral cavity and pharynx | 1 | — | 1 | 0·02 | 0·2 |
| Respiratory system and intrathoracic organs | 2 | 1 | 3 | 0·07 | 0·7 |
| Skin | 2 | — | 2 | 0·05 | 0·4 |
| Unknown | 2 | 2 | 4 | 0·10 | 0·9 |
| Total | 262 | 192 | 454 | 11·34 | 100·0 |

TABLE III.—*Leukaemia classification according to age*

| Type | 0 yrs M | 0 yrs F | 1–4 yrs M | 1–4 yrs F | 5–9 yrs M | 5–9 yrs F | 10–14 yrs M | 10–14 yrs F | Incidence ($10^5$) | % Total |
|---|---|---|---|---|---|---|---|---|---|---|
| Acute lymphoblastic | 2 | 4 | 37 | 27 | 23 | 19 | 7 | 8 | 3·17 | 88·2 |
| Acute myeloid | — | — | 3 | 3 | 2 | 2 | 1 | 1 | 0·27 | 7·6 |
| Other | 1 | — | — | 1 | — | 2 | — | 2 | 0·15 | 4·2 |
| Total | 3 | 4 | 40 | 30 | 25 | 23 | 8 | 11 | 3·60 | 100·0 |
| % Total | 4·9 | | 48·6 | | 33·3 | | 13·2 | | | 100·0 |

TABLE IV.—*Non-Hodgkin's lymphoma and Hodgkin's disease*

| Type | 0 yrs M | 0 yrs F | 1–4 yrs M | 1–4 yrs F | 5–9 yrs M | 5–9 yrs F | 10–14 yrs M | 10–14 yrs F | Incidence ($10^5$) | % Total |
|---|---|---|---|---|---|---|---|---|---|---|
| Diffuse lymphocytic lymphoma | — | — | 4 | 1 | 8 | 1 | 4 | — | 0·45 | 33·3 |
| Histiocytic lymphoma | — | — | — | — | — | — | 1 | 1 | 0·05 | 3·7 |
| Other NHL | — | — | 2 | — | 2 | 3 | 1 | 1 | 0·22 | 16·7 |
| Hodgkin's disease | — | — | — | 1 | 7 | 1 | 14 | 2 | 0·62 | 46·3 |
| Total | — | — | 6 | 2 | 17 | 5 | 20 | 4 | 1·35 | 100·0 |
| % Total | — | | 14·8 | | 40·7 | | 44·4 | | | 100·0 |

TABLE V.—*CNS tumours*

| Type | 0 yrs M | 0 yrs F | 1–4 yrs M | 1–4 yrs F | 5–9 yrs M | 5–9 yrs F | 10–14 yrs M | 10–14 yrs F | Incidence ($10^5$) | % Total |
|---|---|---|---|---|---|---|---|---|---|---|
| Astrocytoma | | | | | | | | | | |
| (Grades I–II) | 1 | 1 | 6 | 1 | 2 | 3 | 2 | — | 0·40 | 17·8 |
| (Grades III–IV) | — | — | 3 | — | 1 | 1 | 2 | 3 | 0·25 | 11·1 |
| Other and unclassified | — | 3 | 2 | 1 | 1 | 1 | 3 | 1 | 0·30 | 13·3 |
| Medulloblastoma | 2 | — | 6 | 3 | 3 | 4 | — | 1 | 0·47 | 21·1 |
| Ependymoma | 1 | 1 | 2 | 2 | 1 | 1 | 1 | 1 | 0·25 | 11·1 |
| Other glioma | 1 | 1 | 3 | 4 | 1 | 5 | 2 | 1 | 0·45 | 20·0 |
| Other | — | 1 | 1 | — | 2 | — | — | 1 | 0·12 | 5·6 |
| Total | 5 | 7 | 23 | 11 | 11 | 15 | 10 | 8 | 2·25 | 100·0 |
| % Total | 13·3 | | 37·8 | | 28·9 | | 20·0 | | | 100·0 |

NHL are 2 cases of Burkitt's lymphoma, both in females. The incidence of NHL and HD appears to be almost equal, but there is a strong male preponderance, with a sex ratio of 3·91 for the entire group, 5·25 for the HD and 3·14 for the NHL. HD tended to occur in older children as compared with non-Hodgkin's lymphoma. Three (12%) of the 25 cases of HD were of the lymphocyte-predominant type, 10 (40%) were of mixed cellularity, 9 (36%) were nodular sclerosis and 3 (12%) were unclassified.

The central nervous system tumours as a whole comprised the largest group of solid tumours (Table V). There were 49 males and 41 females in the CNS cases, a ratio of 1·20. Most of the brain-stem gliomas were diagnosed on the basis of clinical and radiological findings, and were classified as glioma NOS, though in practice they are almost without exception found to be astrocytic. Astrocytomas were classified as low-grade (Kernohan grades I–II) or high-grade (Kernohan grades III–IV). The second commonest type of CNS tumour was the medulloblastoma, followed by the ependymoma. No cases of meningioma were seen. Eight other intracranial tumors involving either the pituitary or the pineal glands are not included in Table V. Only 3 cases of craniopharyngioma were found in the present series. There were 4 tumours classed as pineoblastomas, and one as a pinealoma.

The commonest type of connective tissue tumour was the rhabdomyosarcoma. Of the 14 cases, 9 were classified as

TABLE VI.—*Connective-tissue tumours*

| Type | 0 yrs M | 0 yrs F | 1–4 yrs M | 1–4 yrs F | 5–9 yrs M | 5–9 yrs F | 10–14 yrs M | 10–14 yrs F | Incidence ($10^5$) | % Total |
|---|---|---|---|---|---|---|---|---|---|---|
| Rhabdomyosarcoma | — | 1 | 7 | — | 2 | — | 1 | 3 | 0·35 | 40·0 |
| Infantile fibrosarcoma | 1 | — | — | — | — | — | — | — | 0·02 | 2·9 |
| Osteosarcoma | — | — | — | — | — | — | 1 | 3 | 0·10 | 11·4 |
| Chondrosarcoma | — | — | — | — | 1 | — | — | — | 0·02 | 2·9 |
| Ewing's tumour | — | — | 3 | 3 | 1 | — | 4 | 3 | 0·35 | 40·0 |
| Haemangiopericytoma | — | — | — | — | — | — | — | 1 | 0·02 | 2·9 |
| Total | 1 | 1 | 10 | 3 | 4 | — | 6 | 10 | 0·87 | 100·0 |
| % Total | 5·7 | | 37·1 | | 11·4 | | 45·7 | | | 100·0 |

TABLE VII.—*Wilms' tumour, neuroblastoma, retinoblastoma and miscellaneous tumours*

| Type | 0 yrs M | 0 yrs F | 1–4 yrs M | 1–4 yrs F | 5–9 yrs M | 5–9 yrs F | 10–14 yrs M | 10–14 yrs F | Incidence ($10^5$) | % Total |
|---|---|---|---|---|---|---|---|---|---|---|
| Wilms' tumour | 1 | 1 | 14 | 8 | 3 | 2 | — | — | 0·72 | 22·1 |
| Neuroblastoma and ganglio-neuroblastoma | 6 | 2 | 11 | 9 | 3 | 2 | 2 | — | 0·87 | 26·7 |
| Retinoblastoma | 3 | 2 | 9 | 5 | — | 1 | — | — | 0·50 | 15·3 |
| Miscellaneous | 2 | 3 | 11 | 4 | 6 | 8 | 4 | 9 | 1·17 | 35·9 |
| Total | 12 | 8 | 45 | 26 | 12 | 13 | 6 | 9 | 3·27 | 100·0 |
| % Total | 15·3 | | 54·2 | | 19·1 | | 11·5 | | | 100·0 |

embryonal, 2 as alveolar and 3 unclassified. Ewing's tumour appeared to be commoner than osteogenic sarcoma (Table VI). Six of the 14 cases were under 5 years of age.

Twenty-nine of the 33 renal tumours were Wilms's tumours. The remaining 4 were neuroblastomas which appeared to originate within the kidney. No cases of congenital mesoblastic nephroma were identified. Neuroblastoma was somewhat commoner than Wilms's tumour. There were 32 cases together with 3 cases of ganglioneuroblastoma. There were 20 cases of retinoblastoma of whom 16 were unilateral and 4 bilateral. No cases of the so-called "trilateral" retinoblastoma (Bader *et al.*, 1980) were seen. Further details of these tumours are given in Table VII. The sex ratios were for Wilms's tumour, 1·64, neuroblastoma and ganglioneuroblastoma, 1·69 and retinoblastoma, 1·50.

### DISCUSSION

Considerable help in the planning of the Queensland Childhood Malignancy Registry was obtained from the Manchester Children's Tumour Registry. Much of the information in this report has therefore been presented in a form analogous to that of the recent report from Manchester (Birch *et al.*, 1980). It is believed therefore that some valid comparisons can be drawn with their series, and probably with others such as the Third U.S. National Survey (Young & Miller, 1975). The Queensland Childhood Malignancy Registry meets 2 of the 3 requirements suggested by Young and Miller, namely a population-based registry and complete ascertainment of cases. The third requirement, special pathological review, is being planned. In addition, this registry is equipped to study some aspects of analytical epidemiology of childhood cancer within Queensland. For example, there appear to be important variations in the incidence of some types of childhood cancer in different areas of the state (McWhirter & Bacon, 1980) and the factors influencing such variations are under study.

The paucity of data on childhood cancer incidence in different parts of the world has already been pointed out (Birch *et al.*, 1980) and it is hoped that publication of data such as ours will lead to the establishment of other specialized child-

TABLE VIII.—*Annual age-specific incidence of some tumours (per* $10^5$)

| Type | U.S. whites | Manchester | Queensland |
|---|---|---|---|
| Leukaemia | 4·21 | 3·31 | 3·60 |
| Non-Hodgkin's lymphoma | 0·74 | 0·45 | 0·72 |
| Hodgkin's disease | 0·58 | 0·36 | 0·62 |
| Central nervous system | 2·39 | 2·25 | 2·25 |
| Retinoblastoma | 0·34 | 0·30 | 0·50 |
| Wilms' tumour | 0·76 | 0·51 | 0·72 |
| Bone tumours | 0·56 | 0·48 | 0·47 |
| Neuroblastoma | 0·94 | 0·65 | 0·87 |
| Rhabdomyosarcoma | 0·45 | 0·39 | 0·35 |
| Miscellaneous | 1·48 | 1·30 | 1·24 |
| Total | 12·45 | 10·00 | 11·34 |

hood-cancer registries. Childhood cancer comprises only about 1% of all cancers, and it is therefore not surprising that very few general cancer registries have made special studies of the incidence of childhood cancer within their regions.

Table VIII compares the annual age-specific incidence of some of the types of cancer in Queensland with that in the U.S. and Manchester. The rates given in the 3 series have not been standardized within the age group 0–14 years. It should be noted, however, that the U.S. series does not include histiocytosis X. The overall incidence of childhood cancer in Queensland appears to lie midway between that of Manchester and that of the U.S. The incidence of childhood cancer in the Manchester region ($10{\cdot}0/10^5$ per year) appears to be one of the lowest reported. For example, a rate of $13{\cdot}48/10^5$ was reported from Sweden (Ericsson *et al.*, 1978) and $14{\cdot}28/10^5$ from the province of Torino (Pastore *et al.*, 1979). The low incidence in the Manchester region seems to be mainly accounted for by low incidences of haematological and reticulo-endothelial malignancies, though the incidences of Wilms's tumour and neuroblastoma in Manchester are also apparently lower than in the United States or Queensland. At present, very little is known about the aetiology of childhood cancer, though very few detailed epidemiological studies have been undertaken. Clearly there is a need not only for further detailed epidemiological studies, but also for cooperation between childhood-cancer registries. This would ensure uniformity of reporting so that meaningful comparisons could be made. Although cancer is an important cause of childhood mortality and effects about 1 child in 600 before the 15th birthday, it remains a relatively uncommon condition. Few childhood registries are therefore in a position to carry out independent epidemiological studies which will show statistically significant results.

In general, the incidence of the various types of tumour in Queensland follow the pattern of other comparable regions, but there are some differences which may be of importance. There is a relatively high incidence of acute lymphoblastic leukaemia as compared with other forms of leukaemia. In Queensland it accounted for 88% of all leukaemia compared with 79% in Manchester. The annual incidences of both acute lymphoblastic and acute myeloid leukaemia appear to be higher in Queensland than in Manchester, though there is evidence that the incidence in Manchester is rising (Birch *et al.*, 1979). It is possible that these observations could be explained on the basis of social factors (McWhirter & Bacon, 1980; Hewitt, 1960).

The incidence of non-Hodgkin's lymphoma and that of Hodgkin's disease are very similar to those reported from the United States (Young & Miller, 1975) while those from Manchester are again appreciably lower. In all 3 series, HD is slightly less common than NHL. As might be expected, the HD cases tended to occur mainly in late childhood (Table IV) since this is typically a disease of adolescents and young adults. The distribution of sub-types differed between Manchester and Queensland, with relatively fewer lymphocyte-predominant and relatively more nodular-sclerosis cases in Queensland.

The incidence of CNS tumours is the same in Queensland and Manchester, and there is also remarkably close agreement on the incidence of the various histological types of tumour. The same is mainly true of the U.S. series, except that there are fewer ependymomas, perhaps as a result of a difference in classification.

Wilms's tumour was another instance in which the incidence in Queensland matched that of the United States much more closely than that of the Manchester region. In fact, it appeared to be almost half as common again in Queensland and the United States as in Manchester, seemingly refuting the suggestion that Wilms's tumour incidence does not vary significantly throughout the world (Innis, 1973). Retinoblastoma, especially the unilateral form, occurred more commonly in Queensland than in either Manchester or the United States, but as the Queensland incidence is based on only 20 cases, the difference may not be significant. Bone tumours had a roughly equal incidence in each of the 3 series, but there was an unusual preponderance of Ewing's tumour in Queensland. Ewing's tumour is extremely uncommon amongst negroes (Miller, 1976) but is usually seen rather less frequently than osteosarcoma in white populations.

Included amongst the "miscellaneous" tumours there were only 3 yolk-sac tumours (1 of the pineal region and 2 of the testis). Although malignant melanoma has an extremely high incidence amongst adults in Queensland, there was only 1 case in this series. Only 2 cases were unclassified, of which one was thought to be a form of carcinoma. There were 12 cases of histiocytosis X, and the remainder of the miscellaneous group consisted of about equal numbers of "adult" tumours and rare childhood tumours.

It has been demonstrated in this study that death certificates are apparently not essential in finding cases of childhood cancer, provided that both clinical and pathological records are thoroughly searched. Using this method it is also possible to determine retrospectively the incidence of childhood cancer in a population. It is hoped that these findings will encourage others to determine and publish the. incidence of childhood cancer in various parts of the world. Data from developing countries would be of particular interest.

### REFERENCES

Australian Bureau of Statistics (1976) *Census: Characteristics of the Population and Dwellings in Local Government Areas.*

Bader, J. L., Miller, R. W., Zimmerman, L. E., Champion, L. A. A. & Voûte, P. A. (1980) Trilateral retinoblastoma. *Lancet*, ii, 582.

Birch, J. M., Marsden, H. B. & Swindell, R. (1979) Acute lymphoid leukaemia of childhood in north-west England. *Lancet*, ii, 854.

Birch, J. M., Marsden, H. B. & Swindell, R. (1980) Incidence of malignant disease in childhood: a 24-year review of the Manchester Children's Tumour Registry. *Br. J. Cancer*, **42**, 215.

Ericsson, J. L.-E., Karnström, L. & Mattsson, B. (1978) Childhood cancer in Sweden. *Acta Paediat. Scand.*, **67**, 425.

Hewitt, D. (1960) Geographical pathology of leukaemia in England and Wales. *Acta Unio Int. Contra Cancrum.*, **16**, 1643.

Innis, M. D. (1973) Nephroblastoma: Index cancer of childhood. *Med. J. Aust.*, ii, 322.

McWhirter, W. R. & Bacon, J. E. (1980) Epidemiology of acute lymphoblastic leukaemia in Brisbane. *Med. J. Aust.*, ii, 154.

Miller, R. W. (1976) Etiology of childhood bone cancer: Epidemiologic observations. *Recent Results Cancer Res.*, **54**, 50.

Pastore, A., Anglesio, E., Cappa, A. P. M., Iuliano, F. & Terracini, B. (1979) Epidemiologia dei tumori infantili nella provincia di Torino 1967–74. *Riv. Ital. Ped.*, **5**, 337.

Teppo, L., Salonen, T. & Hakulinen, T. (1975) Incidence of childhood cancer in Finland. *J. Natl Cancer Inst.*, **55**, 1065.

Williams, A. O. (1975) Tumours of childhood in Ibadan, Nigeria. *Cancer*, **56**, 1065.

World Health Organisation (1976) *International Classification of Diseases for Oncology.*

Young, L. C. & Miller, R. W. (1975) Incidence of malignant tumours in U.S. children. *J. Pediatr.*, **86**, 254.